# The role of the primary auditory cortex in the neural mechanism of auditory verbal hallucinations

**DOI:** 10.3389/fnhum.2013.00144

**Published:** 2013-04-24

**Authors:** Kristiina Kompus, Liv E. Falkenberg, Josef J. Bless, Erik Johnsen, Rune A. Kroken, Bodil Kråkvik, Frank Larøi, Else-Marie Løberg, Einar Vedul-Kjelsås, René Westerhausen, Kenneth Hugdahl

**Affiliations:** ^1^Department of Biological and Medical Psychology, University of BergenBergen, Norway; ^2^Division of Psychiatry, Haukeland University HospitalBergen, Norway; ^3^Department of Clinical Medicine, Section Psychiatry, University of BergenBergen, Norway; ^4^Division of Psychiatry, Department of Research and Development, St. Olavs University HospitalTrondheim, Norway; ^5^Department of Psychology, University of LiègeLiège, Belgium; ^6^Department of Neuroscience, Faculty of Medicine, NTNUTrondheim, Norway; ^7^Department of Radiology, Haukeland University HospitalBergen, Norway

**Keywords:** auditory verbal hallucinations, primary auditory cortex, non-clinical, schizophrenia, auditory attention

## Abstract

Auditory verbal hallucinations (AVHs) are a subjective experience of “hearing voices” in the absence of corresponding physical stimulation in the environment. The most remarkable feature of AVHs is their perceptual quality, that is, the experience is subjectively often as vivid as hearing an actual voice, as opposed to mental imagery or auditory memories. This has lead to propositions that dysregulation of the primary auditory cortex (PAC) is a crucial component of the neural mechanism of AVHs. One possible mechanism by which the PAC could give rise to the experience of hallucinations is aberrant patterns of neuronal activity whereby the PAC is overly sensitive to activation arising from internal processing, while being less responsive to external stimulation. In this paper, we review recent research relevant to the role of the PAC in the generation of AVHs. We present new data from a functional magnetic resonance imaging (fMRI) study, examining the responsivity of the left and right PAC to parametrical modulation of the intensity of auditory verbal stimulation, and corresponding attentional top-down control in non-clinical participants with AVHs, and non-clinical participants with no AVHs. Non-clinical hallucinators showed reduced activation to speech sounds but intact attentional modulation in the right PAC. Additionally, we present data from a group of schizophrenia patients with AVHs, who do not show attentional modulation of left or right PAC. The context-appropriate modulation of the PAC may be a protective factor in non-clinical hallucinations.

## Introduction

Auditory verbal hallucinations (AVHs) are the subjective experience of hearing voices speaking in the absence of corresponding physical stimulation. The main body of research on AVHs comes from schizophrenia patients, due to the high prevalence of AVHs in this clinical group. However, AVHs as a symptom are not dependent on the schizophrenia syndrome, as is evident by their occurrence in multiple other diagnostic groups (Larøi et al., 2012), and even in isolation in otherwise mentally healthy individuals (Sommer et al., 2010). A particularly interesting feature of AVHs is their perceptual quality: the experience may be indistinguishable from real voices, as it may have characteristics of a personalized human voice and appear to be originating in the external physical space. Due to this, it has been proposed that the brain regions dedicated to auditory processing are relevant to experiencing hallucinations. This idea is supported by so-called “symptom capture” studies, which attempt to measure brain activity while subjects are experiencing AVHs (Woodruff et al., 1997; Dierks et al., 1999; Shergill et al., 2000). There are findings both from functional magnetic resonance imaging (fMRI) as well as electroencephalography (EEG) which are consistent with the idea that auditory processing areas show elevated activation during AVHs compared to silent rest. Of particular interest is the finding that this hallucination-related activation appears to be present in the primary auditory cortex (PAC) (see Kompus et al., 2011), potentially explaining the realistic nature of the experience. Further, Braun et al. (2003) reported consistency of hallucination modalities with brain lesions in corresponding sensory areas. As deficits in auditory processing have been observed in hallucinating patients, it has been suggested that the auditory processing regions, including the PAC, may be dysfunctional, either due to structural or functional aberrations. As speech processing is performed by the language-dominant, usually left, hemisphere, the efforts to associate sensory processing and AVHs concentrate on the structural and functional integrity of the left-sided auditory cortex. While the primary auditory cortices of both hemispheres process speech stimuli, the left PAC may be particularly implicated due to relationship with higher perceptual processing regions within the left hemisphere (see below).

Theoretical approaches to the role of the PAC in the experience of AVHs may be divided broadly into two categories [see also Waters et al. (2012) for discussion]. First, neurons in the PAC may trigger the AVHs, due to, e.g., spontaneous (i.e., stimulus-independent) activity which may be either (1) quantitatively different from spontaneous activity seen in non-hallucinating individuals (see, e.g., Dierks et al., 1999), or (2) quantitatively similar, but due to some other factor, such as lack of inhibition, able to propagate to higher levels of processing (see, e.g., Hunter et al., 2006; Northoff and Qin, 2011). Second, the PAC may be considered a “receptor” of AVHs, activating in response to input from higher processing regions. In such a view, the PAC may be considered as (1) lacking a critical inhibition to prevent such top-down activation (see, e.g., Friston and Frith, 1995; Ford et al., 2001), (2) lacking appropriate feedforward connections with a “monitoring” mechanism which normally identifies the activation as originating from internal source (see, e.g., McGuire et al., 1995), or (3) possessing disproportionate amount of excitatory links with higher processing regions compared to auditory pathways (see, e.g., Ford et al., 2009). It is important to note that in all of these approaches, the PAC is not considered to be the single cause for experiencing AVHs, but rather one constituent region, possibly providing an explanation for the question why AVHs are experienced as perceptual events, rather than intrusive thoughts or imagery.

Multiple approaches to characterizing the PAC properties are represented in the literature, including measurement of structural properties as well as functional responses. There are studies examining qualitative differences between groups (e.g., comparing the volume of the PAC between hallucinating and non-hallucinating subjects), as well as quantitative differences within a hallucinating group (e.g., analyzing whether the PAC volume predicts hallucination severity).

We consider it timely to review the evidence of the PAC functioning in subjects with AVHs, to evaluate the various theoretical propositions of how the PAC is involved in the experience of AVHs. In the first part of this paper, we selectively review studies examining the properties of the PAC in the context of AVHs. In the second part, we present new data where we probe the functional properties of the PAC using a paradigm which integrates the manipulation of bottom-up, perceptual features of stimulation with top-down, attentional manipulation of the auditory processing. We examine the responsiveness of the PAC in a group of non-clinical hallucinators (NCHs) compared to non-clinical non-hallucinators, and compare these groups with a hallucinating schizophrenia group.

### Auditory verbal hallucinations and the structural features of the primary auditory cortex

**Figure 1:**
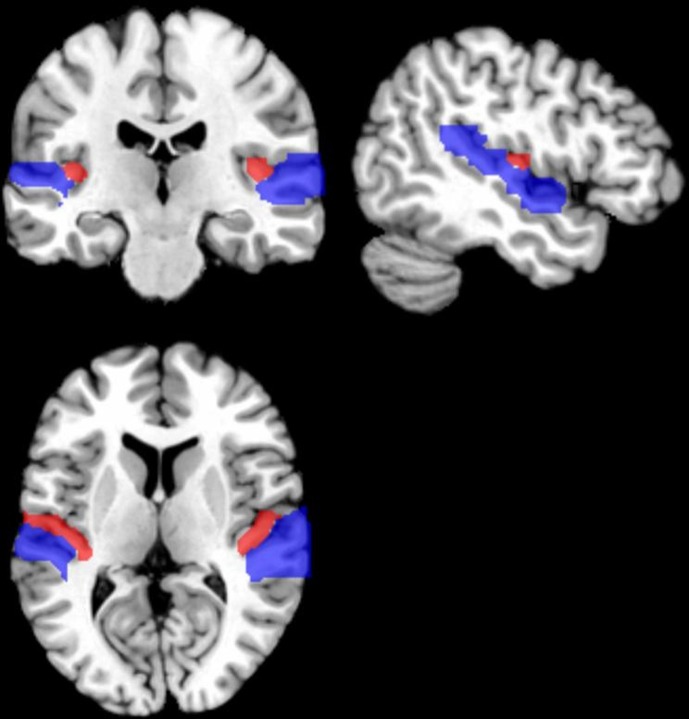
**Schematic illustration of the primary auditory cortex (PAC) on a brain template.** The PAC occupies most of the Heschl's gyrus (red), which extends mediolaterally within the Sylvian fissure. PAC is surrounded by auditory association areas on the superior temporal gyrus (blue).

The PAC, corresponding to cytoarchitectonically defined Brodmann area 41, is located on the transverse temporal gyrus, or Heschl's gyrus (HG), which runs in mediolateral direction within the Sylvian fissure (see Figure 1 for schematic illustration). It is surrounded by secondary auditory processing areas on the superior temporal gyrus (STG). To date, studies on the structural correlates of AVHs have mainly been reported from clinical groups, notably schizophrenia. Post-mortem neuropathological studies of schizophrenia patients have been conflicting regarding specific regional alterations in these patients (Harrison, 1999), however, there is a general consensus in the literature of abnormalities in the STG in schizophrenia (Heckers, 1997), e.g., smaller left-sided volume of the planum temporale (Falkai et al., 1995). Sweet and colleagues have provided a thorough series of reports of post-mortem examinations from the left PAC in schizophrenia patients (Sweet et al., 2003, 2007, 2008; Dorph-Petersen et al., 2007). The changes appear to be concentrated within neuronal layer 3, which gives rise to feedforward projections to auditory association cortices (Douglas and Martin, 2004). In this cortical layer of the PAC, schizophrenia patients show a reduced mean volume (but not number) of pyramidal neurons, reduced dendritic spine density, as well as a reduced density of axon terminals (Sweet et al., 2003, 2007, 2008; Dorph-Petersen et al., 2007). Thus, schizophrenia patients demonstrate abnormalities within the excitatory feedforward circuit of the left PAC. Unfortunately, the clinical profile of the patient groups is not considered in these reports, thus it is not known whether these changes are associated with AVHs.

With the advent of magnetic resonance imaging (MRI), characterization of brain structures *in vivo* has become possible. Volumetric studies of STG frequently demonstrate reduced volume of STG in schizophrenia patients compared to controls, for instance Sun et al. (2009) report 76% of volumetric studies finding a difference between groups. The data is not entirely unequivocal, for instance while some meta-analyses implicate the superior temporal regions to be reduced in volume (Lawrie and Abukmeil, 1998; Wright et al., 2000), a meta-analysis by Vita et al. (2006) found no difference in the temporal lobe structures in first-episode schizophrenia patients. With respect to the PAC in particular, Kasai et al. (2003) showed progressive volume reduction of left HG in schizophrenia patients over time. However, this finding was not correlated to the severity of hallucination symptoms. With respect to schizophrenia patients, the effect of other symptoms, such as thought disorder, may also contribute to regional changes in brain volume and structure (see Shenton et al., 1992; Horn et al., 2010), and the effect may be difficult to distinguish from the effect of hallucinations as a specific symptom.

Volumetric studies may differ from each other considerably with respect to definition of the region of interest, thus the studies using voxel-based morphometry (VBM) to examine regional structural variations may give a more coherent picture. Due to considerable amount of literature from group comparisons using VBM, several meta-analyses have been performed. It must be noted that the meta-analyses of whole-brain data differ from the effect size meta-analyses which are commonly used for other research questions. For MR data, commonly used meta-analysis methods, such as “activation likelihood estimation” (Eickhoff et al., 2005) do not include null-findings, only including studies reporting at least one significant difference anywhere in the brain volume. Thus, it is common for meta-analyses of VBM data to represent the spatial convergence across studies which report significant findings anywhere in the brain, not estimation of population effect size as in a traditional meta-analysis. Multiple meta-analyses have been performed, with three recent studies standing out in their scope. Glahn et al. (2008) performed an anatomic likelihood estimation meta-analysis of VBM studies from schizophrenia patients, including in total 1195 schizophrenia patients (first-episode and chronic) and 1262 healthy controls. A left-sided cluster of convergence was found involving insula, inferior frontal gyrus, precentral gyrus, and STG (including BA 22 and 38). From the report, it is not clear whether the cluster does or does not extend to the PAC. Fornito et al. (2009) used the same meta-analytic approach on a partly overlapping and extended sample of studies, and examined differences in both gray matter concentration as well as gray matter volume. The findings largely converge with the findings reported by Glahn et al. (2008), including a cluster on the left insular cortex which, according to the figure included in the report, appears to spread to left anterior STG. It is not clear whether the cluster does or does not extend to the left PAC. However, a group difference in the right PAC was reported. Bora et al. (2011) used a different meta-analysis method (“signed differential mapping”) to analyze a partially overlapping and extended set of studies, including 1999 schizophrenia patients and 2180 control subjects. Findings show bilateral clusters centered in insula, and extending to the STG. Again, whether the PAC was particularly involved is not explicitly clear in the report, however, according to the figure presenting the results projected on a brain template, the cluster appears to extend to the HG on the right hemisphere.

While the above meta-analyses on schizophrenia are, by integrating data of multiple reports, certainly statistically powerful, they may be not sensitive with respect to AVHs and the PAC since the included individual studies usually pool data from patients that vary with regard to their hallucinatory status. However, it is reasonable to assume that in each of the meta-analyses the schizophrenia sample had higher likelihood of experiencing auditory hallucinations than the control sample, thus the findings are nevertheless relevant. More specific results are reported in two recent meta-analyses, concentrating particularly on the AVHs in schizophrenia patients (Palaniyappan et al., 2012; Modinos et al., 2013). Both of the meta-analyses report data from studies which test the relationship between AVH severity and gray matter volume in VBM studies. Modinos et al. (2013) report a very selective reduction in gray matter volume with increasing hallucination severity, concentrated in the left STG and PAC (size 210 voxels, STG peak coordinates MNI *x*, *y*, *z* = −52, −18, 2, HG peak −46, −14, 6). A trend-level reduction is reported in the right STG and PAC. Palaniyappan et al. (2012) report two clusters, one located in the left insula (size 717 voxels, peak at MNI *x*, *y*, *z* = −42, −4, 2) extending to inferior frontal gyrus and STG at BA 22, the other in right STG (BA 22), extending to right insula (size 318 voxels, BA 22 peak at MNI *x*, *y*, *z* = 58, −6, 10). Thus, although the two analyses broadly agree with bilateral STG being relevant for increased severity of AVHs, they show important divergences in predictors of AVH severity, with Modinos et al. (2013) implicating gray matter loss in bilateral PAC, and Palaniyappan et al. (2012), instead, in bilateral insula as predictors of AVH severity. Consequently, the inferences the authors draw regarding the neural mechanisms of AVHs diverge. Palaniyappan et al. (2012) suggest that insular dysfunction may result in erroneously processing inner speech as external stimulus. Modinos et al. (2013) suggest that “a volumetric abnormality, with the neurons in (STG) being reduced in number and/or spacing or having reduced connectivity […] would block the normal attribution of internal speech.” Considering that the included studies largely overlap, the differences in the results deserve further discussion. While six studies (*n* = 268) are included in both meta-analyses, Modinos et al. (2013) additionally include two further studies (Plaze et al., 2006; van Tol et al., unpublished), resulting in 322 subjects. Palaniyappan et al. (2012) include one further study (Shapleske et al., 2002), resulting in a total of 340 subjects. This study does not report correlational analyses [and is for that reason excluded from Modinos et al. (2013) analysis]. Palaniyappan et al. (2012) report that down-weighting this study did not significantly influence the final results. As the main portion of the subjects overlap, the particular study selection is presumably not the main variable influencing the outcome. The main difference between the two meta-analyses is the analysis method. Palaniyappan et al. (2012) use “signed differential mapping,” which consists of calculating mean effect size across the included studies for each voxel in a brain volume (with the reported peaks being smoothed). Modinos et al. (2013) use a method termed “parametric voxel-based meta-analysis,” which consists of calculating, for each voxel in a brain volume, the proportion of studies which report a significant peak within 10 mm. In both methods, the significance of a voxel is tested against a random spatial distribution of effects. Thus, the interpretation of the findings is different: whereas in the Modinos et al. (2013) study, a significant finding represents a significant proportion of included studies reporting a peak in the immediate neighborhood; in the Palaniyappan et al. (2012) study a significant finding represents a mean effect size across studies. Consequently, one possible (although admittedly speculative) interpretation for the divergence is that the finding of gray matter reduction in insula is less consistent across studies, but if present then at a larger effect-size (in turn suggesting that VBM studies may have lower power specifically at insula); whereas the gray matter reduction in the PAC is consistent, albeit at smaller effect size.

In summary, structural studies offer modest support to the idea that AVHs are associated with structural change in the PAC. To date, the available evidence comes from hallucinations in clinical patients, thus it is not clear whether NCHs demonstrate the same pattern. The reduced gray matter volume in the PAC appears to be more consistent in the right compared to the left hemisphere across the entire schizophrenia population. However, the left PAC gray matter volume reduction appears to be more consistently associated to AVH severity (Neckelmann et al., 2006). There is no well-established theoretical model for the mechanisms of association between progressive loss of the PAC gray matter and progressively more severe hallucinations (as opposed to qualitative differences between groups). Attempts to explain the relationship include suggestions that the structural abnormality may lead to a reduced threshold for neuronal triggering, generating spontaneous neuronal activity in the PAC (Neckelmann et al., 2006). Such explanations necessitate a limit point for gray matter loss to which such an assumption is applicable, otherwise a logical paradox arises; the strongest spontaneous neuronal activation would be predicted in situations where all neurons have decayed.

### Auditory verbal hallucinations and the functional properties of the primary auditory cortex

Functional integrity of the PAC may be probed with functional brain imaging methods, including fMRI and EEG, examining the response of the PAC to external stimulation, as well as with behavioral studies assessing the functional integrity of the PAC.

#### Functional magnetic resonance imaging studies

Recent meta-analyses of fMRI studies examining brain activation to external stimulation in hallucinating subjects indicate that patients with AVHs appear to have reduced fMRI response to external auditory stimuli in the left PAC. Kompus et al. (2011) performed an activation likelihood estimation meta-analysis of 11 studies examining processing of external auditory stimuli, and found convergent reduced activation in the left PAC in schizophrenia compared to control group. Notably, the left PAC was also more activated during “symptom capture” in fMRI studies of ongoing hallucinations (Kompus et al., 2011). A second meta-analysis by Kühn and Gallinat (2012), which did not find convergence in the PAC utilizing a partly overlapping set of studies, is not directly comparable to Kompus et al. (2011) study, as the included contrasts involved a wide variety of conditions (e.g., auditory imagery, speech identity decisions). Ford et al. (2009) reported a region-of-interest (ROI) analysis involving BA 41 responsiveness to auditory stimulation with pure tones in a large sample of patients with schizophrenia and schizoaffective disorder. Hallucinating patients had significantly reduced activation to external sounds in the left BA 41 compared to non-hallucinating patients (with no effect in the right hemisphere). However, this left-sided reduction was not significantly related to the severity of the hallucinations (*r* = 0.02). As the analysis involved a large sample size (66 hallucinating patients) the finding is likely to be reliable.

Considering the relationship between clinical and non-clinical hallucinations, an interesting result was reported by Lewis-Hanna et al. (2011) where individuals prone to sleep-related hallucinations had higher auditory sensitivity, which also co-varied with increased fMRI response to speech stimuli in the left supramarginal gyrus. Further, no differences in the PAC between hallucination-prone and non-prone individuals were reported in tasks examining speech perception and auditory attention. Thus, the functional reduction of the PAC response which is evident in the fMRI studies of schizophrenia patients may not be present in NCHs. This suggestion is supported by Szechtman et al. (1998), who studied a group of subjects susceptible to auditory hallucinations under hypnosis. The hallucination-prone subjects had spatially more extensive activations during auditory stimulation in the superior temporal regions, as measured with positon-emission tomography (PET). However, the individuals described in such studies (sleep-related and hypnosis-related susceptibility to hallucinations) may be a distinct population from NCHs, and thus the relevance of these results to hallucinations in awake state must be interpreted with caution. Compared to persistent non-clinical hallucinations experienced in the awake state, sleep- and hypnosis-related hallucinatory experiences appear to be relatively prevalent in the population and may be predominantly related to sleep disorders (see Ohayon et al., 1996).

To summarize, fMRI response to external auditory stimulation in the left PAC is reduced in hallucinating schizophrenia patients, but the magnitude of reduction does not predict AVH severity. No conclusive data is available for non-clinical hallucinations, but there is a possibility that hallucination-prone healthy individuals do not show reduced activity in the PAC, and may even have more extensive activation to auditory stimulation in other speech-related brain regions. However, the relationship between sleep-related hallucinations and hallucinations in awake state needs to be elucidated further before it can be concluded that such groups are representative of hallucinatory experiences in non-clinical population.

#### Electroencephalographic studies

With regard to EEG and event-related potential (ERP) studies, there are several electrophysiological components originating in the PAC which have been studied in connection with hallucinations (van Lutterveld et al., 2011; Ford et al., 2012). Here, we concentrate on the mismatch negativity (MMN), N100, and auditory steady-state ERP responses.

***Mismatch negativity (MMN).*** MMN is an ERP component, consisting of a reduction in the measured EEG waveform when an auditory stimulus deviates from a train of preceding stimuli in either frequency or duration [see Näätänen (1995) for an overview]. MMN is hypothesized to depend on synaptic plasticity mediated by glutamatergic *N*-methyl-D-aspartate (NMDA) receptors in the primary and secondary auditory cortices (Javitt et al., 1996). Thus, an attenuated MMN may indicate inability to adequately modify synaptic plasticity in response to excitatory neurotransmission resulting from external auditory stimulation. MMN attenuation and its possible predictive validity has been studied in multiple pathologic conditions. Shelley et al. (1991) first demonstrated attenuated MMN in schizophrenia patients. Consistent with one subset of cortical generators of MMN residing in the PAC, Salisbury et al. (2007) demonstrated a negative relationship between MMN amplitude and left hemisphere HG gray matter volume in schizophrenia patients. Importantly, the relationship was evident in both cross-sectional as well as longitudinal examinations. This is in good agreement with Wible et al. (2001), who found a wider extent of fMRI response to duration-deviant stimuli in the left HG in controls than schizophrenia patients. Umbricht and Krljes (2005) performed a meta-analysis of MMN studies comparing schizophrenia patients with healthy controls, and found attenuated MMN in schizophrenia patients (at effect size 0.99, indicating a large effect). The difference between groups was particularly pronounced for duration deviants, but also reliably present for frequency deviants. Umbricht and Krljes (2005) also qualitatively reviewed 22 studies which performed a correlation analysis between MMN attenuation and symptom severity, and noted that the majority of studies did not find a significant relationship. Only three of the 22 reviewed studies reported a significant correlation between positive symptom severity and MMN [note that in one of the three studies, Hirayasu et al. (1998), the effect was found only when appropriate correction for multiple tests was not enforced]. Regarding non-clinical hallucinations, van Lutterveld et al. (2010) found no difference in MMN between a group of non-psychotic individuals with AVHs and a control group. Thus, the existing evidence suggests that glutamate-receptor mediated synaptic plasticity in the PAC, as indexed by MMN, is compromised in schizophrenia patients, but may be not specifically related to AVHs.

***N100.*** The N100 (or N1) ERP component is generated in the PAC in response to onset of external auditory stimulus. It is sensitive to bottom-up features of the auditory stimuli as well as top-down modulations, such as attention (Woldorff et al., 1993). Due to this, it can be seen as an index of successfully engaging in context-appropriate modulation of sensory processing. N100 is generally reduced in schizophrenia patients compared to healthy controls (Rosburg et al., 2008). This is in agreement with the fMRI studies which, as reviewed above, show less activation in the PAC to external stimuli in hallucinating groups. However, the reduction appears to depend on the particular characteristics of stimulus presentation, and does not appear to covary with particular clinical symptoms. Rosburg et al. (2008) note that most studies fail to find associations between N100 amplitude and specific symptoms in schizophrenia patients, with N100 reflecting, at best, general psychopathology load. The results regarding N100 sensitivity to attentional modulation in schizophrenia patients are not completely clear, with some studies suggesting considerable variation resulting from other experimental variables (intensity, stimulation rate) (Baribeau-Braun et al., 1983).

Modulation of N100 response by top-down processes has generated some interesting lines of research in the AVHs field. Hubl et al. (2007) reported reduced N100 in hallucinating subjects to pure tones (sinusoidal tones with 1000 Hz frequency and 70 ms duration) during episodes of AVHs compared to silent rest. This finding is typically interpreted as evidence that AVHs engage the PAC, in direct parallel to non-hallucinating subjects showing reduced N100 amplitude when background noise is present. However, considering the attentional modification being at least partially preserved in schizophrenia, it cannot be excluded that the reduction may also be interpreted as subjects attending the hallucinations at the expense of external stimuli, rather than the PAC representing the sensory features of hallucinations as they unfold.

Ford et al. (2001) have used N100 as a tool to test the theory that the dysfunctional “corollary discharge,” i.e., specific inhibition of auditory cortex to self-generated sounds (see Paus et al., 1996), is associated to hallucination generation. N100 is reduced to self-produced sounds in healthy adults (Martikainen et al., 2005), presumably due to a feedforward model informing the brain of self-initiated actions (Bäss et al., 2008). In case such inhibition of the PAC is dysfunctional, self-generated sounds or inner speech may be perceived as externally originating sounds (Friston and Frith, 1995). Thus, N100 should be reduced in control subjects during their own speech compared to others' speech, whereas it should be equal in schizophrenia subjects. However, evidence is mixed and predictions complicated. For instance, while Ford et al. (2001) demonstrated reduced N100 due to external speech compared to self-generated speech in schizophrenia group (a result not entirely incompatible with the theory), the control group failed to demonstrate any modulation of N100 response between the two conditions, making the finding in schizophrenia group difficult to interpret.

***Auditory steady state response (ASSR).*** Auditory steady state response is a repetitive evoked potential with constant frequency profile, the frequency corresponding to stimulation rate and/or its higher harmonics. (Spencer et al., 2009) showed that in schizophrenia patients, compared to controls, the gamma-band phase locking and evoked power in response to 40 Hz stimulation were overall decreased. However, within the schizophrenia group, both phase locking and evoked power of gamma-band evoked responses at the left PAC source positively correlated with AVHs. A re-analysis of the same data (Mulert et al., 2011) examined the interaction between left and right PAC sources, and found that the inter-hemispheric phase synchronization was positively correlated with AVH symptom scores. The theoretical explanation of such findings is that the PAC shows aberrant oscillatory synchronization, having increased propensity to enter a stable state of oscillatory synchrony independently of external stimulation (Spencer et al., 2009). Koenig et al. (2012) also report the relevance of gamma band synchronization to steady state stimulation to AVHs.

In summary, ERPs offer a mixed view on the functioning of the PAC with respect to hallucinations. While the steady state responses are interesting as they represent a good candidate for neuronal synchronization with the PAC as crucial in the emergence of AVHs, more replications would be desirable. Synaptic plasticity in the PAC, as indicated by MMN, appears to be independent of both the likelihood to experience AVHs (as it is not reduced in NCHs) as well as the severity of AVHs. Similarly, neuronal response to onset of auditory stimuli, the N100 response, does not seem to be specifically modified by experiencing AVHs. Finally, it is noteworthy that a study examining ERPs in non-clinical AVHs (van Lutterveld et al., 2010) found that the non-clinical hallucinators had larger amplitude of one ERP component, namely P300. This component is a positive deflection occurring ~300 ms after the onset of a deviant stimulus, with increased amplitude of P300 reflecting more attentional processing (Picton, 1992). Thus, similarly to fMRI studies, no conclusions can be drawn regarding NCHs; but data suggests that indexes of the PAC function may not differ from non-hallucinating individuals, with some features of auditory processing (localized outside the PAC) showing even enhanced activation.

### Behavioral signs of impaired primary auditory cortex function

Schizophrenia patients often show lower performance on tasks depending on auditory perception, such as tone matching (Rabinowicz et al., 2000). However, overall reduced acuity does not appear to be predominant, as Mathew et al. (1993) reported that control subjects outperformed patients only at frequencies above 1000 Hz. Additional contribution to the reduced performance in auditory tasks appears to be changed laterality pattern, such as found in dichotic listening tasks. In dichotic listening, different input is provided to different ears simultaneously, and the subjects are to report what they hear (Bryden, 1988; Hugdahl, 2003). Relative proportion of reports from left and right ear may indicate temporal lobe functioning, also including the PAC. In right-handed healthy adults, a right-ear advantage for phonological dichotic listening task is typically found (see Ocklenburg et al., 2013). Løberg et al. (2004) reported that AVHs are associated with a reduction in the right-ear advantage. Thus, the left PAC may be compromised with increasing severity of hallucinations in schizophrenia, in agreement with the VBM studies. This relationship has also been observed in a large, multi-center study (Hugdahl et al., 2012).

### Summary

To summarize the literature review, we note that our focus on the PAC in this manuscript does not exclude the importance of higher-order perceptual processing regions, and other brain networks, in the experience of auditory hallucinations. As discussed in the Introduction, the activity of the neurons in the PAC may act as either “triggers” or “receptors” of neural activity related to the experience of hallucinations. In either case, many other brain regions are also implicated in the full experience of hearing a physically non-existing voice speak. This is attested by the wide-spread activation networks observed during the experience of ongoing AVHs, including higher auditory areas in the STG, but also parietal and frontal areas, and subcortical structures (Jardri et al., 2011; Kompus et al., 2011; Allen et al., 2012; see van Lutterveld et al., 2013). The experience of AVHs appears to be initiated as a cascade of activation, spreading along the cortical networks associated with auditory perception, attention and conscious awareness. Interestingly, it has been suggested that the first step in this cascade may be the deactivation of the parahippocampal gyrus (Diederen et al., 2010b), followed by wide-spread activation in temporal, parietal and frontal regions (see also Lennox et al., 1999; Shergill et al., 2004; Hoffman et al., 2008). Due to low temporal resolution of fMRI, the hypotheses of temporal order should be tested with effective connectivity analyses.

## Empirical data

As the literature review presented above shows, most of the studies on properties of the PAC in connection to AVHs are confined to schizophrenia patients. Comparatively little is known about the functionality of the PAC in non-clinical hallucinations. Examining non-clinical individuals who experience hallucinations is of considerable interest, as it allows for “isolation” of the AVHs and examining it separately from any confounding variables such as medication or other psychopathological symptoms. Here, we present an analysis of the functional integrity of the PAC in non-clinical auditory hallucinators from an fMRI project on the neural correlates of non-clinical hallucinations. We used a modulated consonant-vowel dichotic listening task allowing for characterization of the PAC sensitivity to bottom-up saliency (intensity) differences and top-down attentional control, as well as the interaction of these factors.

### Materials and methods

The group of NCHs consists of eight individuals recruited from the general population. Potential subjects were recruited from among the respondents of a population-based study on hallucinatory experiences (Kråkvik et al., in preparation), distributed to 8000 respondents across Norway, randomly chosen from the nationwide citizen register. Additionally, subjects were recruited via advertisements in the local newspaper, and the laboratory website. Screening for voice-hearing was based on a Norwegian translation of the Launay-Slade Hallucination Questionnaire (Bentall and Slade, 1985) items 1 and 2 (hearing voices when no one is around; or hearing own thoughts as voices). Screening also excluded individuals who had visited a physician or a psychologist due to hearing voices. Potential subjects were interviewed with respect to their hallucinatory experiences on the basis of the PSYRATS interview. All subjects presented here reported hearing voices in an awake state, excluding all individuals who had sleep-related hallucinations. In two subjects, the voices consisted of “mumbling” with no clear verbal content, the others reported hearing clear verbal content. All subjects had hallucinatory experiences at least once a month. In two subjects the onset of the voices was within the last 4 years, all others reported onset in childhood or early teenage years. None of the subjects reported taking any medication due to hearing voices, consistent with the screening criterion excluding any potential subjects who had seen a physician due to voice-hearing. Also, no subject reported taking antipsychotic medication at any point during life. The control group consisted of age-matched non-hallucinating individuals recruited from the community. As presented in Table 1, the groups did not differ in mean age or education. All subjects, except one subject in the NCH group, were right-handed as determined with Edinburgh Handedness Inventory. All subjects were native Norwegian speakers. The subjects' hearing threshold was assessed for frequencies 250, 500, 1000, 2000, and 3000 Hz, using the Hughson–Westlake audiometric test (Oscilla USB-300, Inmedico, Lystrup, Denmark). The study was approved by the Regional Committee for Medical Research Ethics in Western Norway (REK-Vest). The subjects gave informed consent prior to participation.

**Table 1:** **Demographic characteristics of the non-clinical hallucinator (NCH) and non-hallucinating non-clinical control group**.

	**NCH**	**Control**
Age, years (SD)^*^	39.3 (12.7)	36.3 (8.9)
Sex (male/female)	3/5	1/7
Handedness (right/left)	7/1	8/0
Education, years (SD)^*^	14.7 (2.3)	16.5 (2.7)
Medication^a^	1/8	0/8
Drugs^b^	2/8	0/8

Note:

* *No significant difference between groups (p > 0.05)*.

a Self-reported use of psychiatric medication within last 12 months (antidepressants, sedatives, other; excluding antipsychotic medication).

b Self-reported use of illicit drugs within last 12 months (cannabinoids, cocaine, LSD, amphetamines, opiates, other).

In order to measure the functioning of the PAC in response to both bottom-up stimulation as well as top-down modulation by attentional demands, we used a consonant-vowel dichotic listening task with attention and inter-aural intensity difference (IID) modification. Detailed description of the task has been provided elsewhere (see Hugdahl et al., 2008; Westerhausen et al., 2010; Falkenberg et al., 2011). In brief, the subjects are presented dichotic pairs of six consonant-vowel pairs (/ba/, /ga/, /da/, /pa/, /ka/, /ta/), spoken by an adult Norwegian male voice with constant intensity and intonation. Only syllables with the same voicing were paired, with stimulus onsets aligned. In each pair, the IID was gradually changed across stimulations to support the perceptual salience of left- and right-ear stimuli. Five levels of IID were used, in steps of 9 dB from strong left-ear preference (18 dB in favor of left ear) to strong right-ear preference (18 dB in favor of right ear). In the condition with 0 IID, the stimuli were delivered at 70 dB sound pressure level; in conditions with variable IID, sound pressure level was kept at 70 dB in the louder ear and reduced in the other ear. To avoid contamination with the MR scanner noise, the stimuli were presented during a silent gap achieved via sparse sampling procedure (see below). Another manipulated factor was attentional instruction. The subjects were instructed to attend and report only from either left- or right-ear stimuli, with the attentional direction alternated between stimulus presentations. The attention instruction appeared 1.5 s before each stimulus. It consisted of a text “attend left/right ear” as well as an arrow pointing in the corresponding direction, and was displayed in goggles mounted to the head coil (NordicNeuroLab, Bergen, Norway). The manipulation with the attention direction, in combination with the IID change, allows this task to be used to flexibly track the neural response to increasingly difficult auditory task (attention and IID favor opposing ears), while keeping constant the type of the of the auditory stimulation (consonant-vowel syllables) (see Hugdahl et al., 2008).

### Data acquisition and analysis

The images were acquired on a GE Signa scanner with field strength 3T. Functional scanning was performed using an echo-planar imaging (EPI) sequence (*TE* = 30 ms; flip angle 90 degrees), acquiring 25 axial slices, covering the cerebral hemispheres and most of the cerebellum. A sparse sampling protocol was used, with a TR of 3.5 s and a TA of 1.5 s, leaving “silent gap” of 2 s between each acquisition, during which the auditory stimulus presentation took place (see Van den Noort et al., 2008).

Behavioral data were analyzed using Statistica software (StatSoft Inc., Tulsa, USA). We examined the auditory acuity using an analysis of variance with factors Group (NCH, control) Frequency (250, 500, 1000, 2000, and 3000 Hz) and Ear (left, right). Dichotic listening behavioral performance data were analyzed using an analysis of variance with the factors Attention (FR, FL), IID (5 levels), Ear (left, right), and Group (NCH, controls). Greenhouse-Geisser correction for degrees of freedom was applied. *Post-hoc* tests were performed using Fisher's LSD procedure. The MR images were analyzed using the Statistical Parametric Mapping (SPM8) software (Wellcome Department of Cognitive Neurology, London, UK) running on Matlab R2010b (Mathworks Inc., Natick, MA, USA). Prior to statistical analyses, the data were preprocessed using the following steps. For each subject, the EPI images were realigned to the first image in the time series and unwarped. The corrected images were normalized to a standard EPI normalization template provided by SPM8 representing MNI space. Finally, the images were smoothed using an 8 mm full-width-at-half-maximum Gaussian filter. For statistical analysis, a general linear model was set up, consisting of ten predictors representing each of the experimental conditions (5 IID conditions and 2 attention conditions), convolved with a canonical hemodynamic response function. A temporal high-pass filter (cutoff at 128 s) was applied. The resulting individual beta images from all subjects were entered into a second-order analysis. The second-order model was set up as repeated measures design, including predictors for each of the experimental conditions, and a group factor. A separate model, which did not remove the within-subject variance, was constructed to examine main effect of group differences. All analyses were restricted to the PAC, with left and right hemispheres tested separately. The region of interest was defined using the SPM Anatomy Toolbox (Eickhoff et al., 2005). The PAC was defined as areas Te 1.0, Te 1.1, and Te 1.2 (Morosan et al., 2001), and saved as anatomical masks for left and right hemispheres separately. The areas involved in the mask are based on cytoarchitectonic studies by (Morosan et al., 2001), and cover the HG from medial (Te 1.1) to central (Te 1.0) and lateral (Te 1.2) portions. The central portion, area Te 1.0, is considered the “core” primary auditory area. The correction for familywise error (FWE) in multiple testing within the region of interest was performed using the SPM routines. The results were thresholded at *p* < 0.05 (FWE). An additional extent threshold of 5 voxels was used to prevent spurious voxels. The MarsBaR toolbox (Brett et al., 2002) was used to extract the parameter estimates from peak voxels to illustrate the direction of results.

## Results

### Auditory acuity

In the analysis of auditory acuity, there was a main effect of Group [*F*_(1, 14)_ = 9.41; *p* = 0.008; η^2^ = 0.14], showing lower acuity for the NCH group. This effect was qualified by a significant interaction between Group and Frequency [*F*_(4, 56)_ = 3.93; *p* = 0.023; η^2^ = 0.1]. *Post-hoc* tests showed that the control group had higher acuity at frequencies 2000 and 3000 Hz, with no significant difference at other frequencies. The Group factor did not interact with any other factor. As possible difference between hearing thresholds in left and right ear is of theoretical interest and relevant to the current paradigm, we performed an exploratory *post-hoc* analysis, testing ear acuities at each frequency step for both groups separately. The control group showed no differences between the ear acuities at any frequency. The NCH group showed higher acuity for left ear at 2000 Hz (*p* < 0.01) and a trend for higher acuity for left ear at 500 Hz (*p* = 0.059).

### Performance of DL task

The results from the three-way analysis of variance are listed in Table 2. As can be seen, there was a trend for a main effect of Group [*F*_(1, 14)_ = 3.26; *p* = 0.092; η^2^ = 0.06], reflecting the tendency for lower overall performance for the NCH group. There were no interactions involving the Group variable. Other effects showed the similar pattern as described in earlier reports (cf. Falkenberg et al., 2011), with a weak trend toward interaction of attention, IID and Ear [*F*_(4, 56)_ = 2.10; *p* = 0.11; η^2^ = 0.02] reflecting how both attention as well as IID influenced the verbal reports. Thus, the NCH group showed a trend toward overall lower number of correctly reported syllables, but no difference from the control group in how attention and IID interact in influencing the performance.

**Table 2:** **Behavioral data analysis for the dichotic listening task for NCH group and control group**.

**Effect**	***F***	η^2^	***p***
Group	3.26	0.06	0.092
Att	2.97	0.00	0.107
Att × Group	0.25	0.00	0.878
**IID**	**7.76**	**0.04**	**0.001**
IID × Group	0.73	0.00	0.516
**Ear**	**11.03**	**0.20**	**0.005**
Ear × Group	0.17	0.00	0.683
**Att** × **IID**	**12.88**	**0.17**	**0.001**
Att × IID × Group	0.87	0.01	0.395
**Att** × **Ear**	**6.23**	**0.05**	**0.026**
Att × Ear × Group	0.49	0.00	0.494
**IID** × **Ear**	**7.34**	**0.15**	**0.001**
IID × Ear × Group	1.32	0.03	0.282
Att × IID × Ear	2.10	0.02	0.108
Att × IID × Ear × Group	0.77	0.01	0.527

Note: Significant findings (*p* < *0.05*) are marked in bold. F, f-statistic; η^2^, effect size; p, significance level.

Results of an analysis of variance, with factors attention instruction (Att), inter-aural intensity difference (IID), Ear, and Group.

### Imaging results

We first evaluated the planned contrast of Attention × IID, to verify whether the previously described modulation by this interaction in the superior temporal areas was present in the current sample. This was found to be the case: the Attention × IID interaction was significant in the PAC in both the left [MNI (*x*, *y*, *z*,): (−54, −24, 12)] as well as right [MNI (*x*, *y*, *z*): (60, −8, 6)] hemisphere. The direction of the interaction agreed with previous findings (Falkenberg et al., 2011), with increased activation when both attention and IID favored the same stimulus. Next, we examined whether this Attention × IID response interacts with the Group factor. There was no significant Attention × IID × Group interaction in left or right PAC, indicating that the NCH group and control group modulated their PAC similarly.

**Figure 2:**
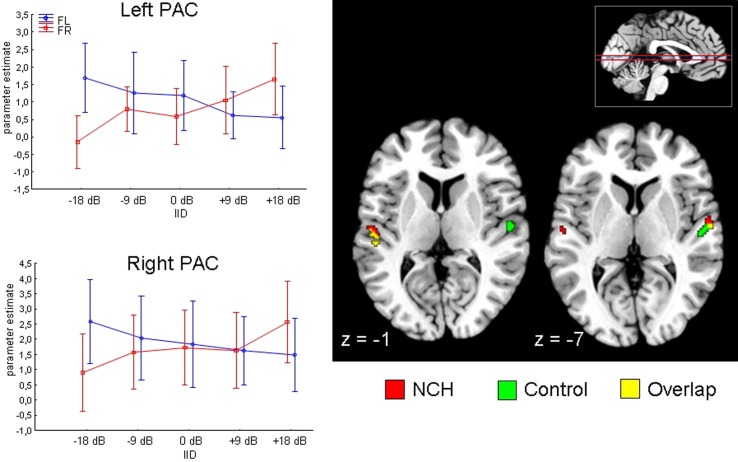
**Attention by IID interaction in the primary auditory cortex (PAC) projected on a brain template.** Two axial slices are presented, at z-coordinate −1 and −7 (position of slice presented with red horizontal lines on inset in the right corner). Results from NCH (red) and control group (green) are plotted separately for illustrative purposes. Line graphs on the left side show changes in parameter estimates (arbitrary units) in the left and right PAC (peak voxel) to change in interaural intensity difference (IID) and attention (FR, forced-right; FL, forced-left), pooled across groups. Vertical lines represent 95% confidence intervals.

We performed a qualitative examination of the Attention × IID response in each group separately, to ensure that the response was in fact present in each group. The corresponding results are presented in Figure 2. As can be seen, both groups presented the bilateral Attention × IID response. There were subtle differences in the distribution of the activation within the subfields of the PAC, with the NCH group showing spread in more lateral direction. For the control group, over 70% of the activated voxels were situated within area Te 1.0 in both the left and right side, with relatively few voxels spreading in lateral direction to area Te 1.2 (left: 3.7%, right: 12.9%). By contrast, in the NCH group the activation was spread more laterally in both hemispheres. On the left side, the majority of the activated voxels were still within Te 1.0 (56.4%), but a larger proportion of the lateral region Te 1.2 was involved (23.0%). On the right side, most of the cluster was in fact situated within Te 1.2 (78.6%), with only 15.6% of the voxels in Te 1.0. As these differences were not strong enough to give rise to an interaction with the Group factor, this lateral shift in the contextual modulation of the PAC response should be considered as a tentative observation.

**Figure 3:**
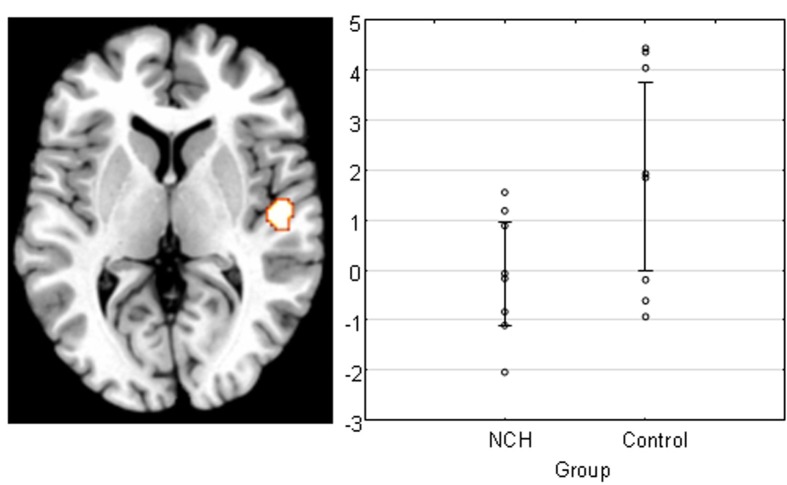
**Main effect of group across all levels of attention and IID in the right PAC projected on a brain template.** Scatterplot shows the individual subjects' mean parameter estimate at peak voxel, demonstrating that the reduction of activation was consistent within NCH group. Lines represent 95% confidence intervals.

Finally, we examined the main effect of Group to see whether activation to the stimulation differed between the groups for the PAC. We found a difference in the right PAC, with the direction showing that the control group activated this area significantly stronger than NCH group (Figure 3). The cluster of activation was almost entirely situated within the area TE 1.0, covering a large portion of it (53.3%).

### Exploratory comparison with clinical hallucinations

For the comparison with clinical hallucinations, we re-analyzed a partial sample from a previous study on schizophrenia patients (Falkenberg et al., submitted) with the same task and imaging parameters. We selected 8 subjects (3 males, 5 females), attempting the best possible age-match to the NCH subjects. The mean age of the final sample was 31.1 (standard deviation 9.9), which did not differ significantly from the NCH and control group [*F*_(2, 21)_ = 1.1; *p* = 0.34]. All subjects were right-handed, and the mean illness duration of the group was 6.9 years. These subjects underwent an auditory task where the attentional demands and IID were manipulated identically to the study described above. However, the functional imaging session for these subjects included a “free-report” baseline task immediately preceding the attention and IID manipulation part. Consequently, as the structure of the data set was different from the NCH study described above, we present these data as a qualitative exploration of the Attention × IID interaction effect in the PAC within a schizophrenia group, rather than a direct statistical comparison of group differences with NCH group. We tested whether the schizophrenia sample showed the Attention × IID interaction in the PAC, as demonstrated in both control subjects as well as the NCH group. We could not find a significant interaction in the PAC in either hemisphere. Only when we exploratively used a non-corrected threshold of *p* < 0.05, small clusters of activation were found bilaterally (peak corrected *p*-value: left 0.18; right: 0.22). Thus, the schizophrenia group showed a qualitative difference in Attention × IID effect in comparison to NCH and control groups.

## Discussion

The results of this empirical study show subtle differences for the NCH group in auditory information processing. First, auditory threshold testing showed lower auditory acuity in the NCH group for high frequencies (2000 and 3000 Hz). Exploratory *post-hoc* tests at each frequency step showed that NCH group had significant ear acuity differences at 2000 Hz, with right ear acuity being lower. The energy of the consonant-vowel syllables used in the current study is predominantly represented within 1000 Hz range. Thus the lower acuity at the affected frequencies should not interfere with the current task. In agreement with this, there were no significant differences in the dichotic listening test performance. While there was a weak trend toward group differences in the overall number of correct reports, the groups did not differ in how the attention and IID influence performance.

In the fMRI data, a significant difference between the NCH and control group was found in the right PAC. This difference was expressed as an overall reduced activation in the right PAC in the NCH group compared to the control group. This difference was confined to area Te 1.0, covering a large proportion of the implicated brain region. This cytoarchitectonically defined area has the widest layer IV of the subfields of the PAC, and receives the majority of the ascending projections from the medial geniculate body in the thalamus (Morosan et al., 2001). Thus, between-group differences in the response of this area to auditory stimulation indicate reduced functionality at the earliest steps of auditory processing. This observation may be interpreted as a parallel to the microstructural alterations in schizophrenia patients, and reduced activation in response to auditory stimulation in hallucinating patients, as discussed above.

In both groups, the bilateral PAC response was sensitive to the interaction between attention and IID. PAC showed increased activation when the attentional direction and IID favored the same ear (i.e., the “stronger” side had to be reported), and decreased activation when the attention and IID were conflicting (i.e., the “weaker” side had to be reported). Such a pattern of combined influences from stimulus features and attention in determining the activation of sensory cortex has been demonstrated in visual attention (Boynton, 2009), and can be interpreted to reflect early attentional modulation effects, e.g., within the biased competition framework (Desimone and Duncan, 1995). As the Attention × IID effect did not interact with the Group factor in either the left or right PAC, and exploratory *post-hoc* tests showed a significant effect for each group, it may be concluded that the NCH group is capable of attentional modulation of the PAC on equal level with the control group. In this, the NCH group shows a qualitative difference from the group of schizophrenia patients who did not show significant Attention × IID modulation in the PAC. A possible interpretation of the data is that a reduction in the PAC activation to external sounds is functionally related to the experience of hallucinations; whereas the preserved ability to modulate the PAC in agreement with the current task set is a protective factor which prevents negative consequences to general functioning. Thus, the current data suggest the possibility that the difference between clinical and non-clinical hallucinations may be expressed as difference in the ability to modulate the brain areas involved in hallucinatory experiences.

An exploratory examination of the spatial features of the Attention × IID effect suggested that there may be slight differences between the groups. In particular, the NCH group showed a lateral spread of the activation into the area TE 1.2, which was particularly pronounced in the right hemisphere. It is possible that the extension of the Attention × IID modulation of the NCH group into the lateral portion of the HG is a consequence of reduced functionality of the central region of the HG. HG demonstrates a “tonotopic” frequency-sensitive gradient, with increasing preference for lower frequencies from medial to lateral direction (Formisano et al., 2003; Humphries et al., 2010; Langers and van Dijk, 2012). Thus, the NCH group may have increased the modulation in the lateral regions to improve sensitivity to the lower-frequency components of the consonant-vowel syllables as a consequence of reduced functionality of the central region.

The main limitation of the current study is the relatively small number of participants. Thus, the negative finding of no difference in attentional modulation of PAC activation should be interpreted with caution due to limited statistical power. The pattern of results from the schizophrenia group, suggests that any dysfunction the NCH may experience in attentional modulation of PAC is not as severe as that observed in schizophrenia population. Due to the small sample size, the present results, albeit consistent with previous literature on the functioning of PAC in subjects with auditory hallucinations, should be considered preliminary, and need to be replicated in larger samples.

Another aspect is the larger number of females among the NCH group (5 subjects), which, considering the overall modest group size, may have influenced the results. We have shown previously that sex differences are not evident in neuronal activation for the type of dichotic listening task used here (Falkenberg et al., 2011; Hirnstein et al., in press), thus we do not consider it likely that the findings presented here are susceptible to sex distribution of the sample. Nevertheless, the trend in the present sample to be female-dominated is similar to other reports of neuroimaging data in NCHs [for example, consider reported male/female ratios such as 3/15 (van Lutterveld et al., 2010), 5/16 (Diederen et al., 2012), 11/24 (Diederen et al., 2010a), 13/22 (De Weijer et al., 2013)], but also in behavioral data. For instance, Sommer et al. (2010) report a sex distribution of 30 men and 73 women in their sample of NCHs. It remains to be examined whether this represents a true sex bias in tendency to experiencing non-clinical hallucinations, or can be explained by other factors, such as willingness to participate in psychiatric studies.

Finally, we note that while the paradigm used here to examine the attentional modulation of PAC has been previously demonstrated to be an effective measure to test the attentional and language networks of the brain (Westerhausen et al., 2010; Falkenberg et al., 2011), it cannot be excluded that it may be relatively less sensitive to subtle changes in the modulation of the PAC by attention. However, as behavioral data from the forced-attention dichotic listening paradigm has shown (Westerhausen and Hugdahl, 2010), a similar type of paradigm is generally sensitive to cognitive dysfunction, including multiple psychiatric conditions, but also, e.g., the effect of sleep deprivation in cognitively healthy young adults.

## Conclusion

The majority of the studies examining the structural and functional properties of the PAC in relation to AVHs reports data from individuals with a schizophrenia diagnosis. Although AVHs are prevalent in this group, comparison with non-clinical, non-hallucinating control group may be confounded by not only other symptoms of schizophrenia, but also antipsychotic medication. In the schizophrenia group, the structural and functional properties of the PAC tend to show a relationship with AVHs, however, the literature is not entirely consistent. Schizophrenia patients have micro-and macrostructural alterations within the PAC, but a large part of the reports does not consider the hallucinatory status of the patients, thus there remains the possibility that these findings may be associated with other symptoms of schizophrenia (such as delusions or cognitive decline), and therefore are not specifically associated with the experience of AVHs. There appears to be a relationship between AVH severity and cortical gray matter volume as measured with VBM, but more confirmatory evidence would be desirable before consensus can be reached. Reduced activation of the PAC in response to auditory stimulation, as measured with fMRI, seems to be characteristic of hallucinating subjects compared to non-hallucinating controls. For ERPs, neither MMN nor N100 appear to be specifically related to AVHs, whereas there are a few interesting reports showing that auditory steady-state response to 40 Hz stimulation is affected by AVHs. Behaviorally, AVHs are associated with reduced right-ear advantage in the consonant-vowel dichotic listening task. Relatively little is known about the functioning of PAC in non-clinical hallucinations. Our findings from a group of NCHs show reduction of the right PAC activation for speech sounds, but (in contrast to the group of schizophrenia patients) preserved modulation by interaction of stimulus properties and attention. It is possible that the context-appropriate modulation of the PAC constitutes a protective factor in distinguishing the non-clinical from clinical hallucinations. The precise characteristics of the PAC properties in relation to AVHs should be studied further, including subjects from clinical as well as non-clinical groups with AVHs, with particular emphasis on the functional and structural connectivity of the PAC, higher-order perceptual processing regions and brain areas providing top-down regulation.

### Conflict of interest statement

The authors declare that the research was conducted in the absence of any commercial or financial relationships that could be construed as a potential conflict of interest.
